# Editorial: Psychosocial Risk Factors in the Development and Maintenance of Eating Disorders

**DOI:** 10.3389/fpsyg.2022.964626

**Published:** 2022-07-13

**Authors:** Matteo Aloi, Gianluca Lo Coco, Antonino Carcione, Giuseppe Nicolò, Giorgio A. Tasca, Cristina Segura-Garcia

**Affiliations:** ^1^Outpatient Unit for Clinical Research and Treatment of Eating Disorders, University Hospital “Mater Domini”, Catanzaro, Italy; ^2^Department of Health Sciences, University “Magna Graecia” of Catanzaro, Catanzaro, Italy; ^3^Department of Psychology, Educational Science and Human Movement, University of Palermo, Palermo, Italy; ^4^Third Centre of Cognitive Psychotherapy—Italian School of Cognitive Psychotherapy (SICC), Rome, Italy; ^5^School of Psychology, University of Ottawa, Ottawa, ON, Canada; ^6^Department of Medical and Surgical Sciences, University “Magna Graecia” of Catanzaro, Catanzaro, Italy

**Keywords:** eating disorders, personality, attachment, metacognition, trauma, risk factor

Eating disorders (EDs) are disabling mental disorders (Treasure et al., 2020) responsible for increased risk of mortality, high rates of years lived with disability, poorer quality of life, higher costs and family burden (van Hoeken and Hoek, 2020).

Researchers and clinicians agree that a multifactorial model that includes predisposing, precipitating and maintenance factors, is the most suitable for explaining the etiopathogenesis of EDs, instead of a single cause model (Fairburn and Harrison, 2003). Although the predisposing factors (i.e. genetic, psychological, environmental) for EDs are typically more common among patients with an ED than in people from general population, they do not necessarily predict the onset of the disorder (Jacobi et al., 2004). However, the more ED risk factors are present, the more likely an individual will develop an ED. Thus, identification of risk factors can help clinicians to determine high-risk groups for targeted interventions, designing prevention program content, and informing public policy (Striegel-Moore and Bulik, 2007). Moreover, research work continuing to examine ED risk factors is warranted given that the COVID-19 pandemic has exacerbated risk for ED pathology and dysfunctional eating behaviors (Fernández-Aranda et al., 2020; Sideli et al., 2021; Linardon et al., 2022). Unfortunately, the investigation of risk factors that may lead to the onset and maintenance of EDs is challenging.

Therefore, the aim of this Research Topic is to deepen our understanding about how individual psychological characteristics can influence EDs, often worsening the clinical course of illness and resulting in less effective treatments. The primary goal of this Research Topic is to bring together researchers and clinicians from different theoretical approach to promote and improve discussion between different models psychopathology in the field of EDs.

It is noteworthy that the majority of contributions (80%, 14 out of 18) to this collection focused on clinical samples of patients with EDs.

In this Research Topic, Muzi et al. evaluated the mediating role of personality variables on pre-treatment ED symptomatic impairment and treatment outcome. Their results exhibited that Clusters A and B of the Shedler Westen Assessment Procedure-200 (Shedler and Westen, 2004) were positively associated with higher levels of overall ED symptomatic impairment at treatment intake and worse therapy outcome, with Cluster B showing an additional association with more severe baseline bulimic symptoms. These findings suggest that personality functioning and disorders may predict baseline symptomatic expression and treatment outcome in EDs.

In line with this, Meneguzzo et al., reported no differences in personality traits due to the presence of any trauma during childhood, but their most striking result was that the early maladaptive schema “disconnection and rejection” appears to be a specific mediator in the relationship between childhood trauma and eating psychopathology. The study by Telléus et al. also focused on traumatic events, and found that 67% of the patients with an ED reported traumatic life events, whereby 19% reporting sexual abuse or negative sexual experiences. However, authors stated that a sexual trauma does not necessarily play a causal role in the onset of EDs.

Three studies provide data on the role of the parents' personality traits and attachment in the EDs. Cortés-García et al. considered that the distal risk influence of insecure attachment to the mother in the onset of ED symptoms might be explained by high clinical perfectionism and low self-esteem, after controlling for depressive symptoms. Monteleone et al. deepened the discussion about the parents' coping strategies with anorexia nervosa (AN) by providing evidence that mothers display higher avoidance and seeking information than fathers. Interestingly, lower illness duration predicted higher collusion with the illness in both parents. Cooperativeness, self-directedness, and harm avoidance positively predicted collusion, parental coercion, and seeking information strategies with some differences between fathers and mothers. In sum, illness duration and personality traits of parents affected the type of parental coping strategies developed to face AN in adolescents. Also about the role of parents, Basile et al. reported that safety/protection, care/nurturance, and emotional expression were the most frequently reported unmet needs within the childhood memories of patients, in particular, with regard to the maternal figure. Besides, mothers were described as more abandoning, but at the same time mostly enmeshed in the relationship with their daughters. Conversely, patients perceived their fathers as more emotionally inhibited and neglecting.

Four studies investigated samples of patients suffering from AN. Ramos et al. explored the clinical utility of considering the illness duration at presentation for treatment in AN and clinical impairment as markers of severity. Clinical impairment seems to be the strongest predictor of poor outcome in terms of eating psychopathology, depressive symptoms, emotion dysregulation, and symptom distress. Weineck et al. evaluated the discrepancy between explicit feelings of power and implicit power motives and its relationship to anxiety. Patients with AN displayed significantly lower explicit feelings of power, however, they showed similar implicit power motives compared to healthy controls (HC). The authors consider that the discrepancy between explicit feelings of power and implicit power motives is related to anxiety and may represent a risk factor to the illness maintenance in AN. Mikhaylova and Dokuka conducted interesting research using a concentric circle methodology analyzing 50 ego networks constructed with data drawn from interviews with Russian-speaking bloggers previously diagnosed with AN that write about this condition. The authors reported that young women with AN tend to have a limited number of social ties, and prefer not to connect with family members. Finally, Leslie et al. evaluated differences in the neural correlates of Theory of mind pathways in young women with AN, and young women weight-restored (WR) from AN, as compared to HC. Their results suggest that the neural processing of theory of mind remains more intact in young women with AN than previously thought.

A stimulating study by Gagliardini et al. explored the role of mentalization in EDs. Through a latent profile analysis, the authors found four different profiles in relation to impairments in the dimensions of mentalization: (1) affective/self/automatic imbalances, characterized by the prevalence of affective mentalization that overwhelms the capacity to reflect on mental states with an imbalance on the self-dimension; (2) external imbalance, characterized by excessive focus on the external cues of mentalization; (3) cognitive/self/automatic imbalances, characterized by over-involvement on the cognitive and self-facets of mentalization, with an impairment in adopting the other mind viewpoint; and (4) cognitive/other/automatic imbalances, indicating those patients who have similar impairments compared to profile 3 but with deficits in self-reflection and an excessive focus on others. These profiles were heterogeneous in terms of EDs represented in each group and were related to significant differences on specific domains such as empathy, attachment style, emotion dysregulation, reflective function, and, interpersonal reactivity.

Another interesting article in our Research Topic concerns the role of emotions and psychophysiological responses across the main EDs diagnosis. French and Chen measured respiratory sinus arrhythmia (RSA), skin conductance (SC), and self-reported emotions at baseline and after film clips that elicited either a neutral, sad, happy, or fearful emotional state. They found that ED groups compared to individuals without EDs generally reported more negative emotions (e.g sadness and anxiety) but were not more emotionally reactive to the film stimuli, which lacked ED-specific content. That is, those with AN reported more fear, those with binge eating disorder (BED) reported more frustration, and those with BED and with bulimia nervosa (BN) reported more tension than healthy controls. Furthermore, patients with BN and BED showed decreased urges to binge during all film clips compared to baseline, suggesting that non-ED specific emotion-eliciting stimuli may at least temporarily decrease urges to binge, even while inducing negative affect.

Gender is a relevant factor in the etiology of ED and the prevalence of the ED show that women are excessively affected, compare to men (Keski-Rahkonen and Mustelin, 2016; Udo and Grilo, 2018). Springmann et al. expand the discussion of gender and ED. They suggest that understanding the role of gender for ED relies on adequate methodological and theoretical access to the construct of gender.

Concerning non-clinical samples, Conceicao et al. evaluated a model representing the interplay between the main aspects of the ED psychopathology and different dimensions of emotion and behavior regulation and self-criticism to understand loss of control (LOC) of eating among 341 college campus students. Their findings showed that LOC of eating occurred for individuals with higher ED psychopathology who experienced depressive symptoms. Moreover, self-criticism was a mediator between emotion regulation and disorder eating, which was significantly associated with LOC eating via increased negative urgency.

Aouad et al. investigated the relationship between defense-style (coping strategies) and ED outcomes over a 5-years period. They found that immaturity and neuroticism but not maturity were the defense-style variables that predicted psychological distress over a 5-years period, while psychological distress predicted only neurotic defense styles. These results may suggest that without treatment, mature, immature and neurotic defense-styles may largely remain immutable to significant shifts over time.

It is well-known that participating in specific activities like dance could be an ED risk factor. Santo André et al. explored how a group of classical ballet dancers perceived their eating attitudes and their bodies, looking for possible EDs symptoms and body image (dis)satisfaction. The constant restrictive diets and other weight-loss strategies to achieve a leaner body were associated with ED symptoms and body dissatisfaction. Remarkably, 50% of the sample were dissatisfied with their current body shape, and for 57% of them the desired body shape was a leaner figure than the one they considered healthy.

Finally, a study explored the psychological correlates of excessive healthy and orthorexic eating in a convenience sample of 399 adults. Strahler et al. found increased difficulties in regulating their emotions and more insecure attachment pattern among people with higher pathological orthorexic eating. According to the authors differentiating between pathological (i.e. clinically relevant) and healthy (i.e. merely a healthy interest in nutrition) orthorexic eating behaviors can be helpful for the diagnosis of orthorexia nervosa.

As an ideal conclusion of the present Research Topic, the article by Pellegrini et al. proposed a reflection on the functioning of EDs that include the impact of early relational trauma on emotion regulation strategies, the role of attachment relationships in the development and maintenance of these disorders, the narrative construction of the self and the symptom, and connections with somatic memories. For a better understanding of eating disorders, they propose the model of Multiple Access Psychotherapy (MAP). MAP focuses on the self-referential modalities through which the individual tends to build the representation of the self and of oneself in the world, adapting the own preferred “access route” to deal with suffering.

The present Research Topic was designed to promote a scientific discussion around, and e fforts to address the complex issue of the psychosocial risk factors of EDs. Relevant manuscripts were collected to increase our knowledge on several aspects of these risk factors in EDs. A major part of clinical research is currently directed toward trying to identify the underlying factors of different psychological disorders, moving beyond any reductionist approach. The identification of such factors provide crucial information for the development of effective prevention and treatment programs.

All the manuscripts in this Research Topic provided a broad description and explanation of the psychosocial risk factors in ED, and sought to provide practical and useful information on their evaluation and usefulness in different treatment options. We believe that identifying risk factors in EDs still represents a challenge for all health and mental health professionals.

Overall, we hope that this Research Topic dedicated to the psychosocial risk factors in EDs will contribute to further improvements in the field, providing interesting insights and help clinician and researchers to grasp the complexity of this topic.

## Author contributions

MA wrote the first draft of the manuscript. CS-G, GLC, AC, GN, and GT provided critical revision of the manuscript and important intellectual contributions. All authors read and approved the submitted version.

## Conflict of interest

The authors declare that the research was conducted in the absence of any commercial or financial relationships that could be construed as a potential conflict of interest.

## Publisher's note

All claims expressed in this article are solely those of the authors and do not necessarily represent those of their affiliated organizations, or those of the publisher, the editors and the reviewers. Any product that may be evaluated in this article, or claim that may be made by its manufacturer, is not guaranteed or endorsed by the publisher.

## References

[B1] Fairburn C. G. Harrison P. J. (2003). Eating disorders. Lancet 361, 407–416. 10.1016/S0140-6736(03)12378-1 12573387

[B2] Fernández-Aranda F. Casas M. Claes L. Bryan D. C. Favaro A. Granero R.. (2020). COVID-19 and implications for eating disorders. Eur. Eat. Disord. Rev. 28, 239–245. 10.1002/erv.2738 32346977 PMC7267370

[B3] Jacobi C. Hayward C. de Zwaan M. Kraemer H. C. Agras W. S. (2004). Coming to terms with risk factors for eating disorders: application of risk terminology and suggestions for a general taxonomy. Psychol. Bull. 130, 19–65. 10.1037/0033-2909.130.1.19 14717649

[B4] Keski-Rahkonen A. Mustelin L. (2016). Epidemiology of eating disorders in Europe. Curr. Opin. Psychiatry 29, 340–345. 10.1097/YCO.0000000000000278 27662598

[B5] Linardon J. Messer M. Rodgers R. F. Fuller-Tyszkiewicz M. (2022). A systematic scoping review of research on COVID-19 impacts on eating disorders: a critical appraisal of the evidence and recommendations for the field. Int. J. Eat. Disord. 55, 3–38. 10.1002/eat.23640 34773665 PMC8646470

[B6] Shedler J. Westen D. (2004). Refining personality disorder diagnosis: integrating science and practice. Am. J. Psychiatry 161, 1350–1365. 10.1176/appi.ajp.161.8.1350 15285958

[B7] Sideli L. Lo Coco G. Bonfanti R. C. Borsarini B. Fortunato L. Sechi C.. (2021). Effects of COVID-19 lockdown on eating disorders and obesity: a systematic review and meta-analysis. Eur. Eat. Disord. Rev. 29, 826–841. 10.1002/erv.2861 34460991 PMC8652707

[B8] Striegel-Moore R. H. Bulik C. M. (2007). Risk factors for eating disorders. Am. Psychol. 62, 181–198. 10.1037/0003-066X.62.3.181 17469897

[B9] Treasure J. Duarte T. A. Schmidt U. (2020). Eating disorders. Lancet 395, 899–911. 10.1016/S0140-6736(20)30059-3 32171414

[B10] Udo T. Grilo C. M. (2018). Prevalence and correlates of DSM-5–defined eating disorders in a nationally representative sample of U.S. adults. Biol. Psychiatry 84, 345–354. 10.1016/j.biopsych.2018.03.014 29859631 PMC6097933

[B11] van Hoeken D. Hoek H. W. (2020). Review of the burden of eating disorders: mortality, disability, costs, quality of life, and family burden. Curr. Opin. Psychiatry 33, 521–527. 10.1097/YCO.0000000000000641 32796186 PMC7575017

